# Molecular Epidemiology of Skin-Dwelling Filariae and Risk Factors for *Mansonella streptocerca* Infection, Gabon

**DOI:** 10.3201/eid3207.251800

**Published:** 2026-07

**Authors:** Capucine Marie Sicard, Mara Fischer, Chiara Wizemann, Marilen Bartling, Linda Martin, Miriam Rodi, Juliana Inoue, Sabrina Valeria Sinopoli, Esther Mehmel, Andrea Kreidenweiss, Pierre Blaise Matsiegui, Michael Ramharter, Selidji Todagbe Agnandji, Jana Held

**Affiliations:** Institute of Tropical Medicine Tübingen, University Hospital Tübingen, Tübingen, Germany (C.M. Sicard, M. Fischer, C. Wizemann, M. Bartling, L. Martin, M. Rodi, J. Inoue, S.V. Sinopoli, A. Kreidenweiss, J. Held); Bernhard-Nocht Institute for Tropical Medicine, Hamburg, Germany (E. Mehmel, M. Ramharter); Centre de Recherches Médicales de Lambaréné (CERMEL), Lambaréné, Gabon (A. Kreidenweiss, M. Ramharter, S.T. Agnandji, J. Held); German Center for Infection Research (DZIF), partner site Tübingen, Tübingen (A. Kreidenweiss, J. Held); Centre de Recherches Médicales de la Ngounié, Fougamou, Gabon (P.B. Matsiegui); German Center for Infection Research, Partner Sites Hamburg-Lübeck-Borstel-Riems, Hamburg (M. Ramharter); Institute for Medical Microbiology, University Hospital Münster, Münster, Germany (S.T. Agnandji)

**Keywords:** parasites, vector-borne infections, *Mansonella streptocerca*, *Onchocerca volvulus*, *Wolbachia*, skin snips, molecular epidemiology, filarial parasites, Gabon

## Abstract

*Mansonella streptocerca* is a species of neglected skin-dwelling filarial nematode parasite with scarce epidemiologic data from Central Africa. We conducted a cross-sectional survey of 1,007 adults from 51 rural and semiurban communities in Gabon to update prevalence estimates and identify risk factors. Molecular analyses by quantitative PCR detected filarial DNA in 18.3% of skin snips; *M. streptocerca* predominated (14.2%), and *Onchocerca volvulus* (3.4%) occurred focally in a single rural area. Blood-dwelling parasite species such as *Loa loa*, *M. perstans*, and *Mansonella* sp. “DEUX” were rarely detected. *M. streptocerca* infection was 4 times more frequent in rural areas than in semiurban areas and independently associated with male sex, urticaria, and poor housing conditions. *Wolbachia* DNA occurred in 28% of *M. streptocerca*–positive samples, suggesting endosymbiosis. Our findings reveal a substantial but overlooked burden of *M. streptocerca* nematodes in Gabon and emphasize the need for integrated surveillance of skin-dwelling filarial infections in Central Africa.

*Mansonella streptocerca* is a species of neglected, skin-dwelling filarial nematode that is transmitted by biting midges of the genus *Culicoides*. *M. streptocerca* is one of the least studied human filariae species; epidemiologic data are scarce compared with other species (*1*,*2*). Although often asymptomatic, infections can cause pruritus, papular dermatitis, or lymphadenopathy (*3*,*4*). Whether this species induces immunomodulatory effects, as for *M. perstans* nematodes, is unclear (*5*). Risk factors for infection with this parasite are unknown.

Central Africa presents a relevant epidemiologic context because of the dense rainforest ecosystem that promotes vector populations and human exposure (*6*,*7*). In Gabon, a country recognized as filarial-endemic, data on *M. streptocerca* nematodes are limited. A few parasitologic surveys in the 1970s and 1980s detected dermal filarial species in several regions (J. Chandenier, doctoral thesis, University of Paris VI, 1983), but large-scale molecular investigations have been lacking since. Coendemic filarial species complicate local epidemiology. *Onchocerca volvulus* nematodes, transmitted by *Simulium* black flies, cause major dermatologic and ocular disease, but data from Gabon remain scarce (*8*–*10*). In contrast to *Simulium* black flies, which require fast-flowing rivers for larval development (*11*), *Culicoides* midges typically breed in moist, organically rich substrates, such as damp soil and decaying vegetation (*12*). Those ecologic differences suggest that environmental determinants of transmission might vary substantially between skin-dwelling filariae. The *Loa loa* nematode, a blood-dwelling species, can occasionally be detected in skin in cases with high parasitemia (*13*,*14*). Loiasis is widespread in Gabon; hyperendemic areas are characterized by high microfilarial loads (*15*–*17*). Its coendemicity with onchocerciasis is programmatically relevant because ivermectin, the standard antiparasitic used for onchocerciasis control, can cause severe adverse events in persons with high *L. loa* microfilaremia (*18*). In addition, other blood-dwelling filaria such as *M. perstans* and the recently described *Mansonella* sp. “DEUX” nematodes are frequent blood parasites in populations in Gabon (*19*,*20*), yet their detection in skin snips is not documented. Among those human filariases, only onchocerciasis is currently included in the World Health Organization (WHO) list of neglected tropical diseases. No evidence of sustained large-scale antifilarial mass drug administration programs in Gabon was identified in the literature; the lack of evidence might partly reflect the difficulty of implementing such programs in the context of limited data on disease distribution and *L. loa* coendemicity (*21*).

Skin snipping has historically been the diagnostic standard of skin-dwelling filariae such as *O. volvulus* and *M. streptocerca*. Molecular tools have improved sensitivity and specificity of skin-snip analysis, enabling simultaneous detection of multiple species and clearer mapping of parasitic distributions.

This study was designed to update prevalence estimates of skin-dwelling filarial infections in 2 provinces of central Gabon, with a particular focus on *M. streptocerca* nematodes. We aimed to establish the risk factors and symptoms associated with this parasite. By combining population-based skin snip sampling with molecular assays and standardized questionnaires, we sought to better characterize the burden of these neglected filariae and inform future control strategies.

## Methods

### Ethics Approval

This cross-sectional study was conducted by convenience sampling during May 2022–December 2024. Ethical approval was granted by the Institutional Ethics Committee of the Centre de Recherches Médicales de Lambaréné (reference no. CEI-001/2022). All participants provided written informed consent before enrollment. Participation was voluntary, and participants were informed of their test results. *Onchocerca*-positive persons were offered doxycycline as treatment. The study followed the International Conference on Harmonization of Good Clinical Practice and the Declaration of Helsinki.

### Study Area and Population

The study took place in Ngounié and Moyen-Ogooué provinces in central Gabon, covering rural and semiurban communities around Lambaréné, Bifoun, Sindara, and Fougamou. Gabon is a country in Central Africa dominated by rainforest ecosystems, providing suitable conditions for filarial transmission.

**Figure 1:**
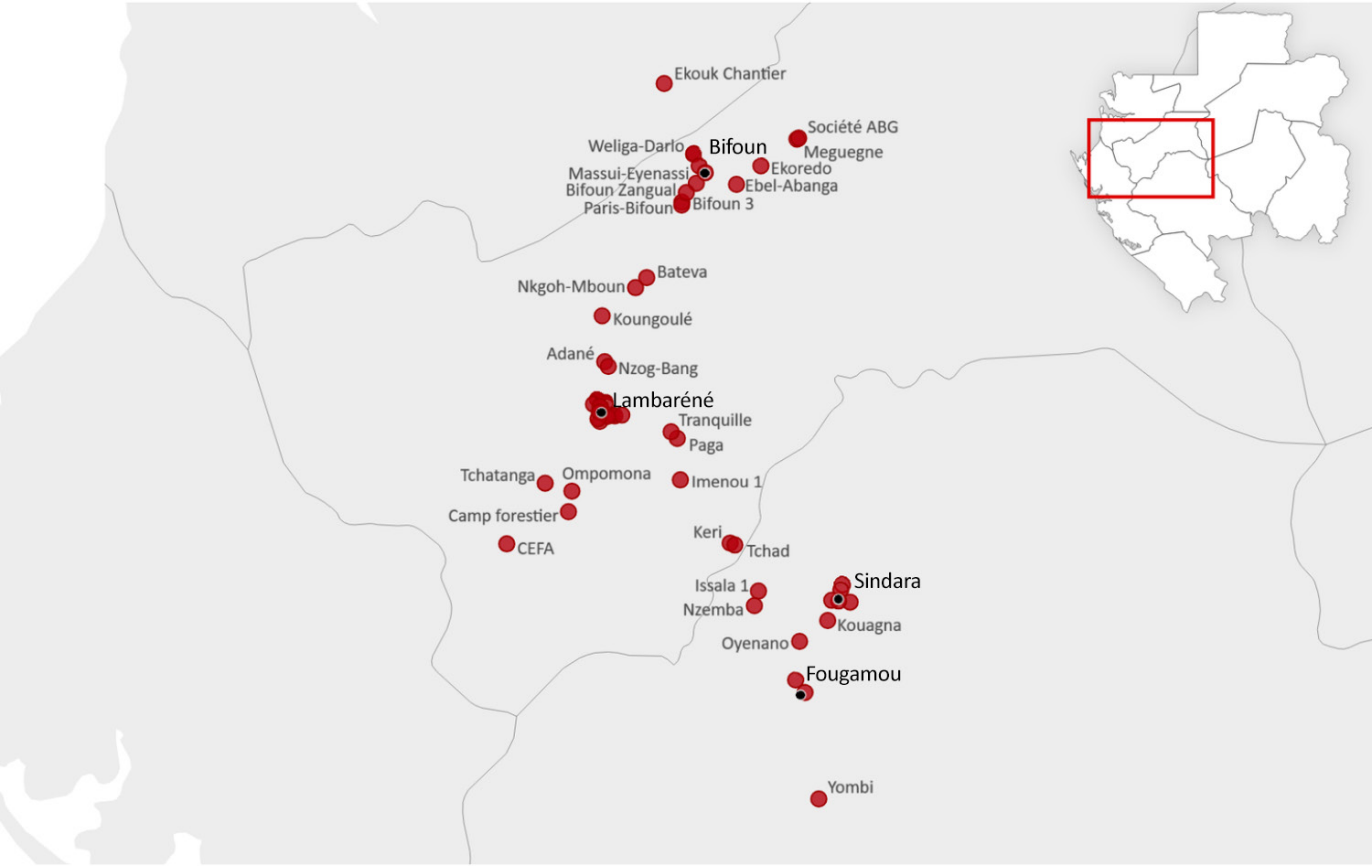
Sampling sites across central Gabon in study of molecular epidemiology of skin-dwelling filariae and risk factors for *Mansonella streptocerca* infection, Gabon. For clarity in labeling, nearby semiurban sampling sites were grouped under their respective town names (e.g., Lambaréné, Bifoun, Fougamou). Although Sindara is a cluster of rural villages, not a town, it was labeled as a town because of the high number of sampling sites in the area and its central role in the analysis. Inset map shows location of study area in Gabon.

Study sites were classified as rural or semiurban. Semiurban sites were the towns of Fougamou, Lambaréné, and Bifoun, defined by population size of >1,400 inhabitants (*22*) and partial access to infrastructure such as healthcare, electricity, or sanitation. Rural sites were small, often isolated villages with limited to absent public services. In total, we sampled 51 locations (35 rural and 16 semiurban) (Figure 1).

Adults (>18 years of age) residing in the area who provided written consent were eligible to participate. Recruitment was community-based and conducted in public space. Each participant received a pseudonymized study identification.

### Sample Collection

We collected a superficial skin snip (≈2 mg) from the scapular region under local anesthesia (EMLA; Aspen Pharmacare, https://www.aspenpharma.com). We took snips using sterile cannulas and scalpels and disinfected wound sites before and after sampling. We stored snips in DNA/RNA Shield (Zymo Research, https://www.zymoresearch.com) at −20°C until further on-site procedures.

### Questionnaire Data

Trained interviewers administered a structured questionnaire in French language. Demographic information included sex, age, occupation, and type of residence. Occupations were grouped into 5 categories: agriculture/forest labor, non-agricultural manual labor, health/public sector, commerce, and other (students, irregular work, unemployment). Vector exposure was assessed by self-reported frequency of bites by *Culicoides* midges (fourous in French) per day (>50, 10–50, <10, or never).

Self-reported clinical data included symptoms associated with filarial infections (pruritus, rash, urticaria, fatigue, joint pain, subcutaneous nodules, stomach pain, headaches); frequency was scored on an ordinal scale (daily, weekly, monthly, never). We recorded eye symptoms (yes/no), uncommon in *M. streptocerca* infections, to explore potential overlap with *O. volvulus* or *L. loa* infections. Participants reported medical history (chronic illness, filarial diagnosis in the past year), recent antiparasitic treatment (Appendix), travel outside their region within 1 year, and presence of domestic animals or rodents in the household. We assessed housing quality with 4 binary indicators: protected drinking water, electricity, cement flooring, and improved sanitation. A composite housing score (0–4) reflected infrastructure quality; higher scores indicated better housing conditions. Participants also rated the effects of skin problems on daily life (not at all, a little, a lot, very much).

### Laboratory Procedures and Quantitative PCR Assay Design

We extracted DNA from skin snips using the Monarch Genomic DNA Purification Kit (New England Biolabs, https://www.neb.com), eluted it in 60 µL buffer, and stored at −20°C until analysis. Filarial DNA detection targeted the internal transcribed spacer 1 (ITS1) region (Appendix Table 1). We performed screening with a published pan-filaria quantitative PCR (qPCR) (*23*) targeting a well-conserved sequence shared by all filarial species. Positive samples underwent a preamplification step of the ITS1 region following established protocols (*21*) to increase template availability for downstream assays.

We then conducted species-specific qPCRs for *M. streptocerca*, *M. perstans*, *Mansonella* sp. “DEUX,” *O. volvulus*, and *L. loa*. Species-specific probes for *M. streptocerca* and *O. volvulus* nematodes were newly designed within the ITS1 region (Appendix). Each 20 µL reaction included buffer (SensiMix II Probe No-ROX [Meridian Bioscience, https://www.meridianbioscience.com] for pan-filaria and *L. loa* qPCR; 10× PCR Buffer [QIAGEN, https://www.qiagen.com] for preamplification; SensiFAST Probe No-ROX Mix [Meridian Biosciences] for all other reactions), primers (Integrated DNA Technologies, https://www.idtdna.com), and species-specific hydrolysis probes (Eurofins Genomics, https://eurofinsgenomics.com). Cycling consisted of an initial denaturation (95°C, 10 min), followed by 45 cycles of denaturation (95°C, 10 s) and annealing/extension (58°C, 60 s). For *Mansonella* sp. “DEUX” and *L. loa*, we performed annealing at 62°C. Positive controls consisted of synthetic plasmids carrying ITS1 inserts of the targeted species (*M. streptocerca*, *M. perstans*, *Mansonella* sp. “DEUX,” *O. volvulus*) (Appendix Table 2). For *L. loa*, we used monoinfected DNA samples with low cycle quantification (Cq) values as controls. Negative controls included nuclease-free water and noninfected human DNA.

We screened a subset of *M. streptocerca*–positive samples for *Wolbachia* DNA by qPCR, according to published protocols (*19*,*24*). Assays targeted the *Wolbachia ftsZ* gene using species-specific primers and probes (Appendix Table 1) after a preamplification step. We selected the *Wolbachia* cell-division gene (*ftsZ*) as a highly conserved marker that enables detection across nematode-associated *Wolbachia* supergroups C, D, F, and J. Each reaction included *O. volvulus* DNA as a positive control and nuclease-free water as a negative control.

We performed all molecular assays in duplicate using a LightCycler 480 system (Roche, https://www.roche.com). Because the ITS1 region is multicopy and a preamplification step has been incorporated, we interpreted the Cq values as relative measures of template abundance rather than as direct estimates of microfilarial density.

### Data Management and Statistical Analysis

We pseudonymized and analyzed all data in R version 4.5.0 (The R Project for Statistical Computing, https://www.r-project.org). We considered a sample positive if amplification occurred in >1 of 2 assay duplicates with a characteristic sigmoidal curve and any Cq value in qPCR.

We calculated prevalence estimates with exact (Clopper-Pearson) 95% CIs. We assessed associations between infection status and categorical variables (binary or multi-category) using the Pearson χ^2^ test of independence (or Fisher exact test when expected counts were <5). We defined age categories (18–34, 35–50, 51–62, >63 years) using quartiles to distribute participants evenly across categories and analyzed them as ordered variables using the Cochran-Armitage trend test to evaluate linear trends in prevalence across categories.

We tested ordinal questionnaire variables (e.g., frequency of reported symptoms, number of *Culicoides* midge bites, housing score) using trend tests or Mann-Whitney U tests as appropriate (Table 1). To account for potential collinearity, we evaluated dependence between key predictors (occupation, housing quality, sex, and rural/urban residence) using χ^2^ tests of independence (Table 1). We interpreted variables showing strong overlap with caution in multivariable models.

**Table 1:** Univariate analysis of associations between demographic, environmental, and clinical variables and *Mansonella streptocerca* infection in study of molecular epidemiology of skin-dwelling filariae and risk factors for *M. streptocerca* infection, Gabon*

Category	No. participants	No. (%)		p value (test)
		*M. streptocerca* qPCR-positive	*M. streptocerca* qPCR-negative	
Total no.	1,007	143 (14.2)	864 (85.8)	
Age, y				
18–34	249	37 (25.9)	212 (24.5)	0.893 (Cochran-Armitage)
35–50	261	34 (23.8)	227 (26.3)	
51–62	249	34 (23.8)	215 (24.9)	
63–100	231	35 (24.5)	196 (22.7)	
Unknown	17	3 (2.1)	14 (1.6)	
Sex				
M	504	102 (71.3)	402 (46.5)	**<0.001** (χ^2^)
F	489	40 (28.0)	449 (52.0)	
Unknown	14	1 (0.7)	13 (1.5)	
Occupation				
Agriculture	447	72 (50.3)	375 (43.4)	**0.007** (χ^2^)
Nonagricultural manual labor	82	19 (13.3)	63 (7.3)	
Health and public sector	86	14 (9.8)	72 (8.3)	
Commerce	49	3 (2.1)	46 (5.3)	
Other	328	33 (23.1)	295 (34.1)	
No answer	15	2 (1.4)	13 (1.5)	
Comorbidities				
Yes	247	28 (19.6)	219 (25.3)	0.199 (χ^2^)
No	739	110 (76.9)	629 (72.8)	
No answer	21	5 (3.5)	16 (1.9)	
No. *Culicoides* midge bites/day				
>50	382	47 (32.9)	335 (38.8)	0.440 (Mann-Whitney U)
10–49	345	51 (35.7)	294 (34.0)	
<10	208	33 (23.1)	175 (20.3)	
Rare/never	47	4 (2.8)	43 (5.0)	
No answer	25	8 (5.6)	17 (2.0)	
Everyday life impacted by skin condition				
A lot	55	3 (2.1)	52 (6.0)	0.410 (Mann-Whitney U)
Sometimes	116	12 (8.4)	104 (12.0)	
Rarely	181	23 (16.1)	158 (18.3)	
Never	330	25 (17.5)	305 (35.3)	
No answer	325	80 (55.9)	245 (28.4)	
History of helminth infection within 1 y				
No	941	130 (90.9)	811 (93.9)	0.509 (χ^2^)
Yes	43	8 (5.6)	35 (4.1)	
No answer	23	5 (3.5)	18 (2.1)	
Anthelminthic treatment intake within 1 y				
No	906	124 (86.7)	782 (90.5)	0.219 (χ^2^)
Yes	77	15 (10.5)	62 (7.2)	
No answer	24	4 (2.8)	20 (2.3)	
Recent travel within 1 y				
Yes	718	96 (67.1)	622 (72.0)	0.377 (χ^2^)
No	273	43 (30.1)	230 (26.6)	
No answer	17	4 (2.8)	13 (1.5)	
Presence of animals inside the habitation				
Yes	813	98 (68.5)	715 (82.8)	**<0.001** (χ^2^)
No	176	42 (29.4)	134 (15.5)	
No answer	18	3 (2.1)	15 (1.7)	
Filaria-related ocular symptoms				
Yes	322	41 (28.7)	281 (32.5)	0.313 (χ^2^)
No	650	100 (69.9)	550 (63.7)	
No answer	35	2 (1.4)	33 (3.8)	
Housing score				
4	133	5 (3.5)	128 (14.8)	**0.011** (Mann-Whitney U)
3	127	10 (7.0)	117 (13.5)	
2	249	23 (16.1)	226 (26.2)	
1	132	21 (14.7)	111 (12.8)	
0	53	4 (2.8)	49 (5.7)	
No answer	313	80 (55.9)	233 (27.0)	

*Bold p values indicate statistical significance (p<0.05). qPCR, quantitative PCR.

We analyzed independent predictors of *M. streptocerca* infection using multivariable logistic regression. We derived a parsimonious final model by stepwise Akaike information criterion selection and formally tested a sex/occupation interaction. We presented results as adjusted odds ratios (aOR) with 95% CIs and p values; we excluded missing responses. Analyses focused solely on *M. streptocerca* nematodes, the predominant species detected in skin snips.

## Results

We enrolled 1,007 adults from 51 sampling points. Participants were balanced by sex (49% female, 50% male) and were 18–97 years of age (Table 2). Most (63.9%) lived in rural areas, whereas 36.1% were from semiurban centers (Table 2). Agriculture and forest-related work predominated in rural villages, whereas semiurban residents were more often employed in commerce, health, or public services (Table 2).

**Table 2:** Population characteristics and prevalence of filarial DNA in 1,007 scapular skin snips in study of molecular epidemiology of skin-dwelling filariae and risk factors for *Mansonella* *streptocerca* infection, Gabon*

Category	No. (%) participants				
	Total	Age, y			
		18–34	35–50	51–62	>63
Total	1,007 (100.0)	249 (24.7)	261 (25.9)	249 (24.7)	231 (22.9)
Sex					
F	489 (48.6)	111 (22.7)	125 (25.6)	129 (26.4)	120 (24.5)
M	507 (50.3)	137 (27.0)	136 (26.8)	120 (23.7)	111 (21.9)
Setting					
Rural	643 (63.9)	142 (22.1)	151 (23.5)	157 (24.4)	180 (28.0)
Semiurban	364 (36.1)	107 (29.4)	110 (30.2)	92 (25.3)	51 (14.0)
Occupation					
Agriculture and forestry	447 (44.4)	76 (17.0)	121 (27.1)	131 (29.3)	118 (26.4)
Nonagricultural manual labor	82 (8.1)	20 (24.4)	32 (39.0)	18 (22.0)	12 (14.6)
Health and public sector	86 (8.5)	14 (16.3)	23 (26.7)	25 (29.1)	24 (27.9)
Commerce	49 (4.9)	9 (18.4)	23 (46.9)	14 (28.6)	3 (6.1)
Other	328 (32.6)	130 (39.6)	61 (18.6)	61 (18.6)	74 (22.6)
qPCR results					
Pan-filaria qPCR positive	184 (18.3)	47 (25.5)	41 (22.3)	47 (25.5)	45 (24.5)
*M*. *streptocerca* qPCR positive	143 (14.2)	37 (25.9)	34 (23.8)	34 (23.8)	35 (24.5)
*Loa loa* qPCR positive	41 (4.1)	9 (22.0)	12 (29.3)	15 (36.6)	5 (12.2)
*O. volvulus* qPCR positive	34 (3.4)	11 (32.4)	4 (11.8)	10 (29.4)	7 (20.6)
*M. perstans* qPCR positive	3 (0.3)	1 (33.3)	0	1 (33.3)	1 (33.3)
*Mansonella* sp. “DEUX” qPCR positive	1 (0.1)	0	0	1 (100.0)	0
Co-infections, >2 species)	38 (3.8)	9 (23.7)	9 (23.7)	14 (36.8)	5 (13.2)

*Totals may not sum to 100% due to missing or unknown responses, which are not displayed in the table. qPCR, quantitative PCR.

Pan-filaria screening identified filarial DNA in 18.3% (median Cq 34.42) of skin snips. *M. streptocerca* was the most frequent nematode species at 14.2% (median Cq 19.38), followed by *O. volvulus* at 3.4% (median Cq 17.35). Blood-dwelling nematode species were occasionally detected in skin samples: *L. loa* in 4.1% (median Cq 22.80), *M. perstans* in 0.3% (median Cq 33.21), and *Mansonella* sp. “DEUX” in a single case (0.1% [median Cq 13.32]); 3.8% of the participants were co-infected with >2 species (Appendix Table 3, Figure 1). Of 67 monoinfected *M. streptocerca* nematode–positive samples, 19 (28.4%) tested positive for *Wolbachia* nematodes after preamplification. Median Cq values of *M. streptocerca* ITS1 qPCR were significantly lower in *Wolbachia*-positive samples than in *Wolbachia*-negative samples (12.77 vs. 18.20 by Wilcoxon rank-sum test; p<0.001), which is consistent with higher template abundance in *Wolbachia*-positive samples (Appendix Figure 2). However, given the preamplification step before the species-specific detection, Cq values should not be interpreted as direct quantitative measures of microfilaremia. To validate qPCR results, we sequenced the *Wolbachia ftsZ* target region in 11 samples and confirmed the expected sequence in 9 of them. We generated a consensus sequence from 4 *M. streptocerca* nematode–positive samples (GenBank accession nos. PZ173978–81) (Appendix). Compared with the published *Wolbachia ftsZ* sequence from *M. perstans* (GenBank accession no. KJ631375), the 438-bp consensus sequence obtained from *M. streptocerca* nematode–positive samples showed 8 nt differences.

**Figure 2:**
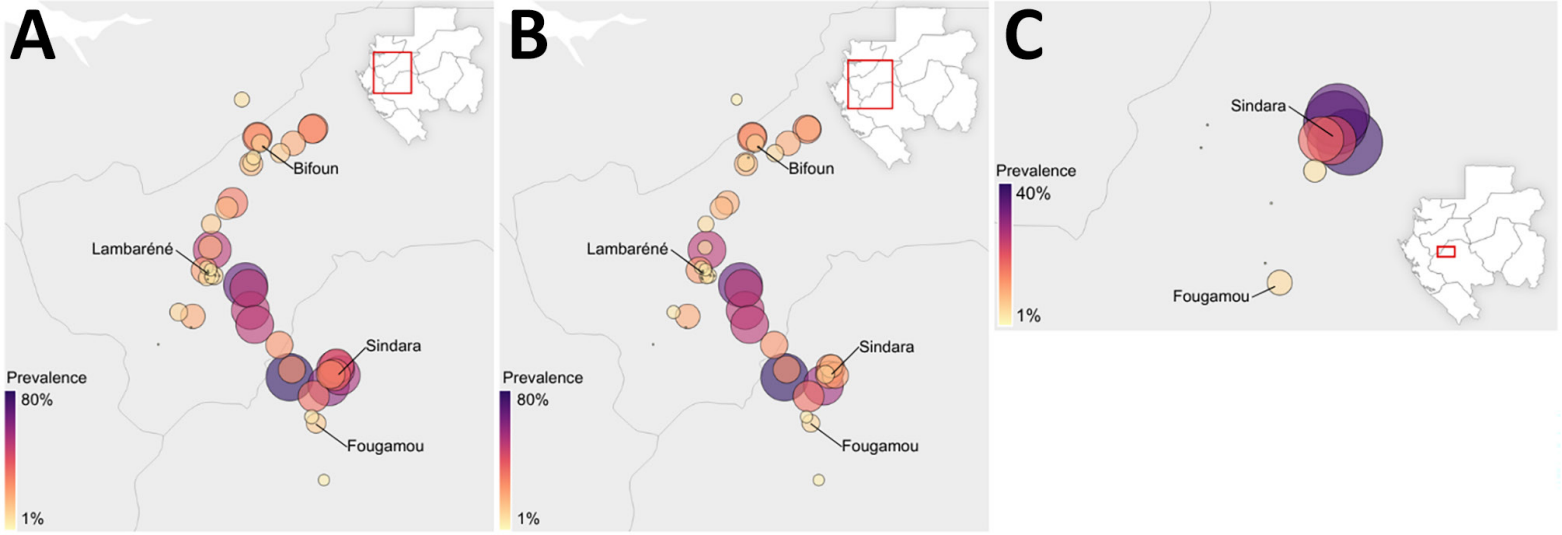
Geographic distribution of skin-dwelling filariae in central Gabon in study of molecular epidemiology of skin-dwelling filariae and risk factors for *Mansonella streptocerca* infection, Gabon. A) Overall filarial DNA prevalence; B) distribution of *M. streptocerca* nematodes; and C) distribution of *O. volvulus* nematodes, showing a focal hotspot around Sindara. Each circle represents 1 sampling area; size and color correspond to prevalence. Inset maps show locations of corresponding area in Gabon.

Geographic mapping showed marked heterogeneity; filaria prevalence ranged from no cases to >60% for different sites (Figure 2, panel A; Appendix Table 4). The distribution of *M. streptocerca* nematodes (Figure 2, panel B) largely mirrored overall positivity, exceeding 40% in some rural sites and reaching up to 80% in Nzoghe-Bang. By contrast, some semiurban sites had no cases (e.g., Atongowanga). *M. streptocerca* nematode prevalence was 19.3% in rural areas (range 0%–80%) compared with 5.2% in semiurban areas (range 0%–23.1%), a highly significant difference (p<0.0001 by χ^2^ test). Infection was 4 times more likely in rural residents (odds ratio 4.34 [95% CI 2.63–7.16]); *O. volvulus* nematodes (Figure 2, panel C) displayed a highly focal pattern, confined almost entirely to 1 area (Sindara area).

Infection was significantly more common in men (102/504 [20.2%]) than in women (40/489 [8.2%]) (p<0.0001 by χ^2^ test) (Table 1). No significant linear trend was observed across age categories (p = 0.893 by Cochran-Armitage test). Occupational categories showed heterogeneous risks: nonagricultural manual laborers had the highest prevalence (19/82 [23.2%]) of the occupational categories, and that association was significant in univariate analysis (p = 0.0065). Agricultural workers had a slightly higher prevalence than the general population (72/447 [16.2%]), although that difference did not reach statistical significance. Housing conditions were also associated with infection. Participants with lower housing scores, reflecting limited access to electricity, water, cement flooring, and sanitation, were more frequently infected (p = 0.0105), suggesting that improved infrastructure exerted a protective effect. Presence of animals inside the household had a protective effect against *M. streptocerca* infection; infected persons were less likely to report cohabitation with pets or domestic animals (p<0.0001). Other variables, including underlying conditions, travel history, recent antiparasitic treatment, and number of *Culicoides* midge bites reported per day, were not significantly associated with infection status.

Self-reported symptoms were examined for associations with *M. streptocerca* infection (Table 3). Joint pain, subcutaneous nodules, urticaria, and stomach pain were more frequent in infected participants, whereas no differences in fatigue, rash, and headaches were noted. Eye symptoms were included as exploratory variables but did not differ by infection status.

**Table 3:** Frequency of reported symptoms by infection status in study of molecular epidemiology of skin-dwelling filariae and risk factors for *Mansonella streptocerca* infection, Gabon*

Symptoms	Total	No. (%) participants								p value
		Ms+ 4	Ms+ 3	Ms+ 2	Ms+ 1	Ms– 4	Ms– 3	Ms– 2	Ms– 1	
Urticaria	976	57 (41)	211 (25.2)	17 (12.2)	150 (17.9)	15 (10.8)	106 (12.7)	50 (36)	370 (44.2)	**0.003**
Fatigue or exhaustion	971	40 (28.8)	160 (19.2)	20 (14.4)	203 (24.4)	17 (12.2)	142 (17.1)	62 (44.6)	327 (39.3)	0.727
Cutaneous rash	971	32 (22.9)	117 (14.1)	7 (5)	113 (13.6)	16 (11.4)	97 (11.7)	85 (60.7)	504 (60.6)	0.396
Headaches	978	48 (34.3)	152 (18.1)	14 (10)	226 (27)	26 (18.6)	180 (21.5)	52 (37.1)	280 (33.4)	0.266
Stomach pain	971	32 (23)	85 (10.2)	12 (8.6)	116 (13.9)	22 (15.8)	171 (20.6)	73 (52.5)	460 (55.3)	**0.018**
Joint pain	977	66 (47.5)	325 (38.8)	34 (24.5)	152 (18.1)	21 (15.1)	115 (13.7)	18 (12.9)	246 (29.4)	**<0.001**
Subcutaneous nodules	947	23 (16.7)	46 (5.7)	48 (34.8)	128 (15.8)	4 (2.9)	45 (5.6)	63 (45.7)	590 (72.9)	**<0.001**

*Ms+, PCR-positive for *M. streptocerca* (n = 143); Ms–, PCR-negative for *M. streptocerca* (n = 864). Numbers 1–4 indicate frequency of symptoms: 4, every day; 3, once a week; 2, once a month; 1, rarely/never. Bold p values indicate statistical significance (p<0.05).

Multivariable logistic regression identified 3 independent predictors (Table 4). Male sex remained strongly associated with infection (aOR 2.9 [95% CI 1.6–5.4]; p = 0.001). Housing score also retained significance; higher scores indicated protection (aOR 0.78 per unit increase [95% CI 0.61–0.98; p = 0.037). Urticaria frequency remained independently associated with infection (aOR 1.35 per category [95% CI 1.10–1.76]; p = 0.009). Among occupational groups, only the assigned group of other occupations showed lower odds than agriculture, whereas we detected no significant sex–occupation interaction (p = 0.24). Because the group was highly heterogeneous and the overall occupation variable did not remain significant after adjustment, that finding is unlikely to represent a true occupational effect.

**Table 4:** Multivariate logistic regression analysis of potential risk factors associated with *Mansonella streptocerca* infection in study of molecular epidemiology of skin-dwelling filariae and risk factors for *M. streptocerca* infection, Gabon*

Predictor	Category (reference)	Adjusted OR (95% CI)	p value	
Sex	Male (female)	2.86 (1.57–5.38)	**<0.001**	
Occupation	Nonagricultural manual labor (agriculture)	1.36 (0.59–3.00)	0.458	
	Health/public (vs Agriculture)	1.31 (0.45–3.37)	0.599	
	Commerce (agriculture)	0.81 (0.19–2.52)	0.747	
	Other (agriculture)	0.41 (0.19–0.82)	**0.014**	
Housing score (0–4)	Per 1-unit increase†	0.78 (0.61–0.98)	**0.037**	
Urticaria frequency (1–4)	Per 1-category increase	1.39 (1.10–1.76)	**0.009**	
Stomach pain (1–4)	Per 1-category increase	0.8 (0.58–1.12)	0.195	
Joint pain (1–4)	Per 1-category increase	1.03 (0.82–1.31)	0.780	
Subcutaneous nodules (1–4)	Per 1-category increase	1.07 (0.76–1.52)	0.693	
Animals in the household	Yes (no)	1.16 (0.49–2.74)	0.730	

*OR, odds ratio. †For housing, adjusted OR <1 indicates that higher housing scores are protective (lower odds of infection). Bold p values indicate statistical significance (p < 0.05).

To explore those confounding patterns, we tested collinearity between key predictors. Strong associations between occupation and both rural/urban residence (p<0.0001) and sex (p<0.0001) were demonstrated by χ^2^ tests of independence. Housing score was strongly correlated with place of residence (p<0.0001); poorer housing conditions were more common in rural areas. The small variation of housing scores by sex (p = 0.003) likely reflects differences in the sampling distribution between rural and semiurban households, rather than a true sex-related difference in housing conditions. Those results confirm that occupation and housing quality overlap strongly with structural demographic variables, which likely explains why occupation did not remain significant in the multivariable model. By contrast, housing retained an independent effect, indicating that even within rural or semiurban settings, better housing infrastructure conferred some protection.

## Discussion

This cross-sectional study shows that nearly 1 in 5 adults in central Gabon carried skin filariae; *M. streptocerca* nematodes dominated. Detected in 1 of 7 participants, *M. streptocerca* nematodes accounted for most filarial infections in scapular skin snips. Prevalence was highly uneven, exceeding 70% in some rural villages but remaining low in semiurban sites. This heterogeneity is consistent with earlier studies conducted in Gabon and Uganda where prevalences ranged from 5% to 90% depending on the region (*4*; J. Chandenier, doctoral thesis, University of Paris VI, 1983). By contrast, *O. volvulus* nematodes were confined to an epidemiologic hotspot (Sindara). As expected, blood-dwelling filariae such as *L. loa*, *M. perstans*, and *Mansonella* sp. “DEUX” were rarely identified. Detection was probably caused by a small amount of blood contaminating the snip during skin sampling or unusual localization of *L. loa* nematodes in the skin (*13*). Those data update the scarce epidemiologic information for skin-dwelling filaria for Gabon (J. Chandenier, doctoral thesis).

The predominance of *M. streptocerca* nematodes confirms the species’ central role in filarial infections in rainforest regions of Central Africa (*3*,*4*). Infection prevalence was clearly influenced by setting, being almost 4 times higher in rural communities than in semiurban communities. Rural residents engage more often in forest-based activities and live in less-protected housing, conditions favoring exposure to *Culicoides* midges. Semiurban environments, by contrast, may reduce risk through fewer vector breeding sites. The *C. grahamii* midge is the only species so far confirmed as a vector (*25*).

The Sindara cluster of *O. volvulus* nematodes illustrates the focal nature of onchocerciasis transmission, linked to the breeding ecology of *Simulium* black flies (*26*). Outside that hotspot, prevalence was negligible, highlighting the need for updated fine-scale mapping in Gabon, where only a few recent studies have addressed the epidemiology of onchocerciasis (*21*,*27*). The focal detection of *O. volvulus* nematodes is consistent with the heterogeneous distribution of onchocerciasis previously reported in Gabon (*21*; J. Chandenier, doctoral thesis). Although *O. volvulus* nematodes were detected in a highly focal pattern restricted to the Sindara region, their presence has programmatic implications. One in 5 persons in Sindara carried *O. volvulus* nematodes, and even a limited cluster of onchocerciasis represents a substantial reservoir. Given the ongoing efforts of the WHO toward onchocerciasis elimination in Africa, such foci could threaten progress, especially if infected persons travel to areas where transmission is already under control (*28*,*29*).

Male sex emerged as one of the strongest predictors of *M. streptocerca* infection; men were >3 times as likely to be infected. This pattern is consistent with reports for other filarial parasites (*30*,*31*). Occupational exposure, outdoor activity, and less frequent use of protective clothing could explain some of the difference in filarial species prevalences between men and women (*32*,*33*), although biologic susceptibility has also been proposed (*16*,*34*). Occupation itself appeared associated in univariate analysis but was strongly correlated with sex and rural residence.

Housing quality was also independently protective. Higher housing scores, reflecting better infrastructure such as cement flooring, electricity, and improved sanitation, were linked to lower infection risk. Although housing remained independently associated with infection, the underlying mechanism is unclear. Better housing might reduce exposure to biting vectors, but housing quality might also reflect differences in the surrounding environment that influence vector abundance and transmission risk.

We observed an unexpected protective association between *M. streptocerca* infection and the presence of animals inside the household. That association might reflect a dilution effect if local *Culicoides* midges also feed on animals, although unmeasured confounding factors cannot be excluded. This finding should be interpreted with caution.

The absence of an association between self-reported number of *Culicoides* midge bites and infection status should also be interpreted cautiously. This variable might have been difficult for participants to estimate reliably in a setting with frequent biting exposure. In addition, *Culicoides* midge bites are nonspecific and might have been difficult to distinguish from mosquito bites or other pruritic skin conditions.

Clinical manifestations were limited, but urticaria remained significantly associated with infection after adjustment. That finding echoes previous descriptions of dermatological involvement (*3*,*4*). Other symptoms, including joint pain, nodules, and stomach pain, did not remain predictive.

We detected *Wolbachia* DNA in approximately one quarter of *M. streptocerca* monoinfected samples. Those endosymbionts are essential for the survival and reproduction of many filarial species, including *Wuchereria bancrofti*, *O. volvulus,* and *Mansonella* species (*M. perstans, M. ozzardi*, *Mansonella* sp. “DEUX”), but not *L. loa* (*35*). Low *Wolbachia* abundance in microfilariae is common; thus, samples with low microfilarial densities might approach the PCR detection limit, as reported previously (*19*,*24*). This finding suggests the presence of *Wolbachia* in *M. streptocerca* nematodes and the therapeutic relevance of doxycycline, which has proven effective in other filarial infections (*36*–*38*).

The first limitation of this study is that scapular skin snips might underestimate *O. volvulus* nematode presence compared with iliac crest samples (*39*). The cross-sectional design cannot capture temporal trends, and symptom data were self-reported because no medical examination was conducted as part of this study. In addition, convenience sampling without random selection might have introduced selection bias and limits the generalizability of our findings. *M. rodhaini* nematodes, previously reported in the region (J. Chandenier, doctoral thesis), were not included in our molecular assays because of lack of DNA sequence data. Last, because our study did not include children or adolescents, we could not assess early-life exposure. However, the similar prevalence across adult age groups suggests that infection is likely acquired in childhood or adolescence and persists over time.

In conclusion, our results confirm that *M. streptocerca* nematodes are highly prevalent but neglected in Gabon, and risk for infection is shaped by ecologic and socioeconomic factors. The protective role of better housing highlights the value of structural improvements within integrated control strategies. Urticaria was the only consistent clinical correlate, warranting further study. The focal detection of *O. volvulus* nematodes underlines the need for updated surveillance and entomological studies to be in line with the WHO goals on onchocerciasis control and elimination. Because most medical consultations in Gabon concern infectious diseases (*40*), addressing *M. streptocerca* and *O. volvulus* nematodes within national control frameworks could reduce their hidden burden. Operational research is needed to evaluate feasible interventions and guide integration into the broader skin-NTD agenda (*41*). Future work should clarify the clinical significance of *M. streptocerca* nematodes, particularly dermatologic and immunomodulatory effects, and their vectors and transmission hotspots.

Appendix Additional information about molecular epidemiology of skin-dwelling filariae and risk factors for *Mansonella streptocerca* infection, Gabon
