# Supplementary material for: Molecular Epidemiology of Skin-Dwelling Filariae and Risk Factors for Mansonella streptocerca Infection, Gabon
Source: Emerg Infect Dis. 2026 Jul;32(7):1123–32. doi: 10.3201/eid3207.251800 (PMC13322432; doi:10.3201/eid3207.251800)
Supplement: Appendix — Additional information about molecular epidemiology of skin-dwelling filariae and risk factors for Mansonella streptocerca infection, Gabon [file 25-1800-Techapp-s1.pdf]

Article DOI: https://doi.org/10.3201/eid3207.251800

EID cannot ensure accessibility for supplementary materials supplied by authors. Readers who have difficulty accessing supplementary content should contact the authors for assistance.

Molecular Epidemiology of Skin-Dwelling Filariae and Risk Factors for *Mansonella streptocerca* Infection, Gabon

Appendix

Supplementary Methods

Design and Validation of Species-Specific ITS1 qPCR Assays

Species-specific hydrolysis probe assays for *Mansonella streptocerca* and *Onchocerca volvulus* were developed within the Internal Transcribed Spacer 1 (ITS1) region amplified during pan-filarial screening.

For *M. streptocerca*, probe design was based on the only publicly available ITS1 sequence at the time of analysis (GenBank accession no. KR868771). For *O. volvulus*, six ITS1 sequences available in GenBank (AF228566, AF228567, AF228572, AF228573, LC850258, EU272179) were retrieved and aligned to generate a consensus sequence.

Multiple sequence alignments including ITS1 sequences of co-endemic filarial species (*M. streptocerca*, *O. volvulus*, *Loa loa*, *M. perstans*, and *Mansonella* sp. "DEUX") were performed using Geneious Prime (Biomatters Ltd, New Zealand). Candidate probe regions were selected to maximize inter-species nucleotide divergence while remaining conserved within the target species. Particular attention was given to incorporating discriminatory mismatches relative to sympatric filarial species.

In silico specificity was evaluated using BLASTn against the NCBI nucleotide database to exclude significant homology with non-target filarial species or human DNA.

Assays were tested and optimized using synthetic plasmids containing ITS1 inserts of the respective species, for which the sequences are provided in Appendix Table 2. For the *O. volvulus* assay, optimization was performed using DNA from an adult worm sample, but for subsequent screening of field samples, synthetic plasmids served as positive controls. Plasmid constructs were serially diluted to assess analytical sensitivity and determine consistent detection across dilution ranges. Final optimized assay conditions are summarized in Appendix Table 1. Reaction conditions (annealing temperature, primer and probe concentrations) were optimized empirically before application to field samples.

Recent Antiparasitic Treatment

The variable "recent antiparasitic treatment" was included because recent drug exposure may influence microfilarial detection. Although no mass drug administration campaigns targeting filarial infections have been conducted in the study area, short courses of antihelminthic drugs (such as mebendazole or albendazole) may be prescribed in local health facilities for nonspecific symptoms such as abdominal pain or diarrhea without any parasitological confirmation. In addition, antiparasitic treatment may occur through self-medication or the use of over-the-counter products. Such short-course treatments are unlikely to affect adult filariae but may transiently reduce circulating microfilariae, potentially influencing molecular detection in skin or blood samples. For this reason, recent antiparasitic treatment was recorded and considered in the analysis.

Supplementary Sequence 1

Partial *Wolbachia ftsZ* consensus sequence (438 bp) obtained from 4 *M. streptocerca*-positive samples:

GCGGGAATGGGTGGTGGCACTGGAACAGGTGCAGCGCCGGTAATTGCAAAA
GCAGCAAGAGAAGCAAAAACCGTAGTTAAGGATAAAGCATTAAAAGAAAAAAAGA
TATTGACTGTTGGAGTTGTAACCAAGCCATTTGGCTTCGAAGGTGTGCGTCGTATGC
GCATTGCAGAGCTTGGACTTGAGGAACTACAGAAATACGTGGATACTCTCATTGTTA
TTCCAAACCAAAACTTGTTTAGAATTGCCAATGAGAAAACTACATTTTCTGATGCAT
TTAAACTTGCTGATAATGTCCTACATATTGGTATCAGAGGAGTAACTGACTTAATGG
TCATGCCTGGGCTCATTAATCTTGATTTTGCTGATATAGAAACAGTGATGAGCGAAA
TGGGAAAAGCAATGATTGGTACTGGAGAAGCGGAGGGAGAAGATAGA

Appendix Table 1. List of oligonucleotide reagents with sequences, melting temperatures, and fluorophores used for molecular detection (qPCR and preamplification) of filarial DNA.

Target (Gene)	Oligonucleotide ID	Sequence (5′-3′)	Length (bp)	Tm (°C)	GC (%)	Adaptation	Reference
Pan-filaria (ITS1)	Pan-Filaria Fwd	CCTGCGGAAGGATCATTAWC	20	57.3	50		(*1*)
	Pan-Filaria Rv	ATCGACGGTTTAGGCGATAA	20	56	45		(*1*)
	Filaria PreAmp Rv	TCGCACTATTTATCGCAGCTAG	22	58.43	45.45		(*2*)
	Pan-Filaria Probe	AGACACCAACGAATATCACCG	21	57.9	47.6	5′-FAM, 3′-BHQ1	(*1*)
Mansonella sp. and *O. volvulus* (ITS1)	MansoOncho Fwd	CGGAAGGATCATTAWCGAGCTT	22	58.4	45.45		(*2*)*
	MansoOncho Rv	CGAATATCACCGTTWATTCAKTTKT	23	56.4	32		(*2*)*
	M. perstans Probe	GCAACATGCATGAGTATATACATAT	25	55.48	32	5′-HEX, 3′-BHQ1	(*2*)
	M. streptocerca Probe	ACATGCATATAGTTGTTATTAGTGAAT	27	55.6	25	5′-ROX, 3′-BHQ2	Current study
	Mansonella sp. 'DEUX' Probe	CTGTATGTATATAGTTGCTTTGCTATT	27	56	26	5′-Cy5, 3′-BHQ1	(*2*)
	O. volvulus Probe	CGAAAAAGAATGTGTTAATAATAGATG	27	55.9	25	5′-FAM, 3′-BHQ1	Current study
L. loa (ITS1)	*Loa loa* Fwd	TGATGATGATATATGATGAAGAAAC	25	53.12	28		(*2*)
	Loa loa Rv	TAGCTAAAATGCTTATTAAGTCTAC	25	53.03	28		(*2*)
	Loa loa Probe	CGCCTAACCGTCGATAACAATG	22	59.8	50	5′-HEX, 3′-BHQ1	(*2*)
Wolbachia (*ftsZ*)	*Wolbachia* ftsZ PreAmp Fwd	ATYATGGARCATATAAARGATAG	23	56.7	28.3		(*3*)
	Wolbachia ftsZ PreAmp Rv	TCRAGYAATGGATTRGATAT	20	56.8	32.5		(*3*)
	Wolbachia ftsZ Fwd	ACRGCRGGAATGGGTGGTG	19	68.2	63.2		(2)
	Wolbachia ftsZ Rv	TTTGYAATTCYTCAAGTCCRAG	22	61.6	38.6		(2)†
	Wolbachia ftsZ Probe	GTTGTAACCAAGCCATTTGGCT	22	65.3	45.5	5′-FAM, 3′-BHQ1	(2)

*Adapted to include detection for *O. volvulus*.
†In Sandri et al., this sequence was reported as template-strand instead of a reverse complement.

Appendix Table 2. ITS1 insert sequences used in pUC57-BsaI-Free synthetic plasmids as positive controls for qPCR assays

Species	Sequence	Length (bp)
M. streptocerca	GGTGAACCTGCGGAAGGATCATTAACGAGCTTCGAAACAAATACATAATAACAATGTAAATGTTATCCATAATTATTATTACTATTCACTTTTATTAGCAACATGCATATAGTTGTTATTAGTGAATAATTAAATAATAATTGATACAACTGAATTAACGGTGATATTCGTTGGTGTCTATACTTTATCCAAATTATCGCCTAAACCGTCGATAATGATGAAGATAAAGCGATAGCTTAATTAAAATTAATTAAGTAGACTTAATAAGCA	270
Mansonella sp. "DEUX"	CGGAAGGATCATTAACGAGCTTCAAAACAAACACAAAATAACAATGAAATGTTATCCATAATTATTATTACTATTCACTTTTATTAGCAACATGTATGAATATATACTGTATGTATATAGTTGCTTTGCTATTATTTAATATTAGTGAATAGTTAAATAATAATTGATACAACTGAATTAACGGTGATATTCG	193
M. perstans	CGGAAGGATCATTAACGAGCTTCCAAACAAATACATAATAACAATGAAATGTTATCCATAATTATTATTACTATTCACTTTTATTTAGCAACATGCATGAGTATATACATATATATAGTTGCTTTGCTATTATTTAATATTAGTGAATAGTTAAATAATAATTGATACAACTGAATTAACGGTGATATTCG	191
O. volvulus	CGTAGGTGAACCTGCGGAAGGATCATTATCGAGCTTCAACAACAACAATAATCATACATATTTTTATTATATGTAATGATCATTATTACCAACCATATCACTTAAATCATTATCATTATTATTATTTTATATTAACATAAAATTTCTTTTTCATTTAAGCAACGAAAAAGAATGTGTTAATAATAGATGAATGATAATAATAGTGATATATTTTGTGATTGGTTAATTAAAAATGAATAAACGGTGATATTCGTTGGTGTCTATACTTTATCCAAGTTATCGCCTGACCGTCGATAACAATGAAGATAAAGCGATAGCTTAATTAATTTTAAGTAGACTTAATAAGCATTTTAGCTAGTATGCTGCCAACAAAACAAATACACACACAAACTATATGTATTTGATTCAATTTTTCATTATTAACATTTTTTTTTAACTCTTAGCGGTGGATCACTTGGCTCGTGGATCGATGAAGAACGCAGCTAGCTGCGATAAATAGTGCGAATTGCAGACG	514

Appendix Table 3. Detailed distribution of filarial coinfections detected in scapular skin snips (n = 1007).

Number of species	Species combination	Total, n (%)
2	*M. streptocerca* + *L. loa*	18 (1.8)
	M. streptocerca + *O. volvulus*	11 (1.1)
	O. volvulus + *L. loa*	5 (0.5)
	M. streptocerca + *Mansonella* sp. “DEUX”	1 (0.1)
	O. volvulus + *M. perstans*	1 (0.1)
3	*M. streptocerca* + *M. perstans* + *L. loa*	1 (0.1)
	M. streptocerca + *O. volvulus* + *L. loa*	1 (0.1)

Appendix Table 4. Detailed distribution of filarial species detected in scapular skin snips per setting (n = 1007).

Setting	Location name	GPS coord. (lat, long)	Total, n	Filaria+	Ms+	Ov+
Bifoun rural	Bifoun 3	−0.305096, 10.372225	10	1 (10.0%)	1 (10.0%)	0 (0.0%)
	Darlo	−0.2164467, 10.3938439	10	3 (30.0%)	3 (30.0%)	0 (0.0%)
	Ebel-Abanga	−0.2723682, 10.4731823	75	9 (12.0%)	7 (9.3%)	0 (0.0%)
	Ekoredo	−0.2380192, 10.5182967	15	3 (20.0%)	3 (20.0%)	0 (0.0%)
	Ekouk Chantier	−0.0859004, 10.3395858	29	2 (6.9%)	1 (3.4%)	0 (0.0%)
	Massui-Eyenassi	−0.27030207, 10.3989207	10	0 (0.0%)	0 (0.0%)	0 (0.0%)
	Meguegne	−0.1887789, 10.5848487	11	3 (27.3%)	3 (27.3%)	0 (0.0%)
	Paris Bifoun 2	−0.310391, 10.371981	17	3 (17.6%)	3 (17.6%)	0 (0.0%)
	Société ABG	−0.1868454, 10.5881418	24	7 (29.2%)	5 (20.8%)	0 (0.0%)
	Weliga Darlo	−0.2155140, 10.3944685	28	7 (25.0%)	7 (25.0%)	0 (0.0%)
	Bifoun Zangual	−0.287532, 10.380531	16	1 (6.2%)	0 (0.0%)	0 (0.0%)
Bifoun semi-urban	Bifoun center	−0.2377785, 10.4041355	11	1 (9.1%)	1 (9.1%)	0 (0.0%)
	Bifoun center 2	−0.2506831, 10.4155886	7	0 (0.0%)	0 (0.0%)	0 (0.0%)
Fougamou rural	Douani	−1.044522, 10.682955	13	7 (53.8%)	3 (23.1%)	5 (38.5%)
	Igono	−1.0123649, 10.6690597	17	7 (41.2%)	3 (17.6%)	6 (35.3%)
	Issala I	−1.024098, 10.513575	12	3 (25.0%)	3 (25.0%)	0 (0.0%)
	Kouagna	−1.078577, 10.641537	25	15 (60.0%)	14 (56.0%)	1 (4.0%)
	Lasong	−1.021554, 10.665868	14	7 (50.0%)	4 (28.6%)	5 (35.7%)
	Nzemba	−1.050967, 10.506083	4	2 (50.0%)	2 (50.0%)	0 (0.0%)
	Oyenano	−1.117101, 10.589618	3	1 (33.3%)	1 (33.3%)	0 (0.0%)
	Sindara 2	−1.042373, 10.661683	29	8 (27.6%)	3 (10.3%)	6 (20.7%)
	Sindara 1	−1.041224, 10.649016	51	18 (35.3%)	13 (25.5%)	9 (17.6%)
	Yombi	−1.408304, 10.624967	28	1 (3.6%)	1 (3.6%)	0 (0.0%)
Fougamou semi-urban	Fougamou	−1.1884252, 10.5822016	137	8 (5.8%)	6 (4.4%)	0 (0.0%)
	Ngouassa	−1.211527, 10.5996092	40	5 (12.5%)	4 (10.0%)	2 (5.0%)
Lambaréné rural	Adané	−0.6, 10.229556	33	6 (18.2%)	2 (6.1%)	0 (0.0%)
	Bateva	−0.4445868, 10.3072796	16	5 (31.2%)	3 (18.8%)	0 (0.0%)
	Camp Forestier	−0.8772663, 10.1624766	3	0 (0.0%)	0 (0.0%)	0 (0.0%)
	CEFA	−0.9366601, 10.0483871	14	0 (0.0%)	0 (0.0%)	0 (0.0%)
	Imenou I	−0.818512, 10.369305	4	2 (50.0%)	2 (50.0%)	0 (0.0%)
	Keri	−0.935315, 10.461293	12	3 (25.0%)	3 (25.0%)	0 (0.0%)
	Koungoulé	−0.5152504, 10.2248792	25	3 (12.0%)	2 (8.0%)	0 (0.0%)
	Nkgoh-Mboun	−0.4631538, 10.2866206	6	1 (16.7%)	1 (16.7%)	0 (0.0%)
	Nzoghe-Bang	−0.6088188, 10.2362963	5	4 (80.0%)	4 (80.0%)	0 (0.0%)
	Ompomona	−0.8392345, 10.1688622	30	6 (20.0%)	6 (20.0%)	0 (0.0%)
	Paga	−0.74189, 10.36369	14	7 (50.0%)	7 (50.0%)	0 (0.0%)
	Tchad	−0.938780, 10.469977	4	2 (50.0%)	2 (50.0%)	0 (0.0%)
	Tchatanga	−0.8245983, 10.1194580	20	2 (10.0%)	1 (5.0%)	0 (0.0%)
	Tranquille	−0.729648, 10.352297	16	11 (68.8%)	11 (68.8%)	0 (0.0%)
Lambaréné semi-urban	Petit Paris 2	−0.69828226, 10.2428391	29	3 (10.3%)	2 (6.9%)	0 (0.0%)
	Petit Paris 3	−0.6999211, 10.2481923	3	0 (0.0%)	0 (0.0%)	0 (0.0%)
	PK5	−0.6986386, 10.2611506	15	0 (0.0%)	0 (0.0%)	0 (0.0%)
	Moussamoukoukou	−0.6698111,10.2160594	19	1 (5.3%)	1 (5.3%)	0 (0.0%)
	Lalala	−0.7104510, 10.2205501	10	0 (0.0%)	0 (0.0%)	0 (0.0%)
	Isaac	−0.7011438, 10.2348885	16	1 (6.2%)	1 (6.2%)	0 (0.0%)
	Chateau	−0.7057743, 10.2170518	12	1 (8.3%)	0 (0.0%)	0 (0.0%)
	Dakar	−0.6876315, 10.2227049	9	0 (0.0%)	0 (0.0%)	0 (0.0%)
	Fangui	−0.6787047, 10.2088094	13	3 (23.1%)	3 (23.1%)	0 (0.0%)
	Hopital Schweitzer	−0.677111, 10.230819	24	1 (4.2%)	1 (4.2%)	0 (0.0%)
	Adouma	−0.681619, 10.221126	16	0 (0.0%)	0 (0.0%)	0 (0.0%)
	Atongowanga	−0.6928928, 10.2193259	3	0 (0.0%)	0 (0.0%)	0 (0.0%)

Filaria+ = Participants who tested positive for filarial DNA; Ms+ = tested positive for *M. streptocerca* DNA; Ov+ = tested positive for *O. volvulus* DNA.

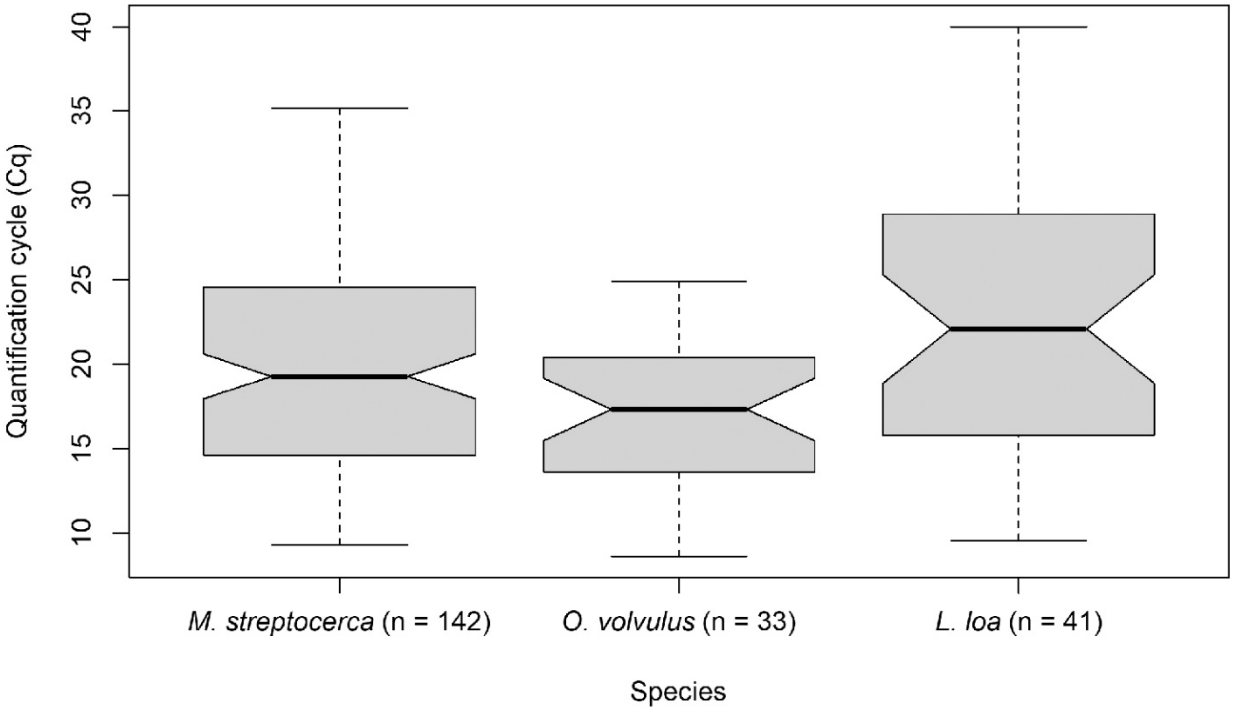

Appendix Figure 1. Distribution of quantification cycle (Cq) values by species detected in skin snips. Boxes indicate the interquartile range, the center line indicates the median (19.38, 17.35 and 22.80 for *M. streptocerca*, *O. volvulus* and *L. loa* respectively) and whiskers indicate the range. Cq values represent the mean of duplicate reactions for each sample. Species with fewer than 10 positive samples (*M. perstans* and *Mansonella* sp. “DEUX”) are not displayed.

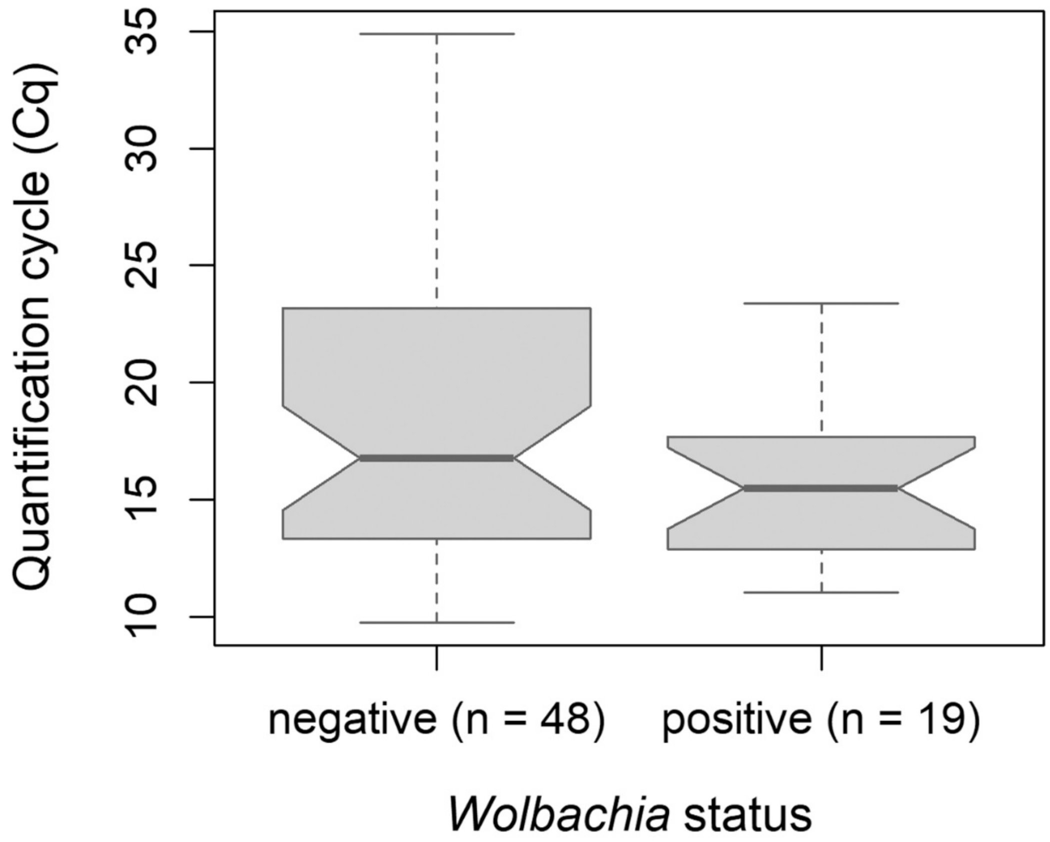

Appendix Figure 2. Distribution of Cq values for *M. streptocerca* by *Wolbachia* status. Boxplots are based on the mean Cq of duplicate reactions for each sample. Boxes indicate the interquartile range, the center line indicates the median (12.77 for *Wolbachia*-positive versus 18.20 for *Wolbachia*-negative), and whiskers indicate the range.

References

1. Bassene H, Sambou M, Fenollar F, Clarke S, Djiba S, Mourembou G, et al. High prevalence of *Mansonella perstans* filariasis in rural Senegal. Am J Trop Med Hyg. 2015;93:601–6. PubMed https://doi.org/10.4269/ajtmh.15-0051

2. Sandri TL, Kreidenweiss A, Cavallo S, Weber D, Juhas S, Rodi M, et al. Molecular epidemiology of *Mansonella* species in Gabon. J Infect Dis. 2021;223:287–96. PubMed https://doi.org/10.1093/infdis/jiaa670

3. Gehringer C, Kreidenweiss A, Flamen A, Antony JS, Grobusch MP, Bélard S. Molecular evidence of *Wolbachia* endosymbiosis in *Mansonella perstans* in Gabon, Central Africa. J Infect Dis. 2014;210:1633–8. PubMed https://doi.org/10.1093/infdis/jiu320
